# The effectiveness of ethno-specific and mainstream health services: an evidence gap map

**DOI:** 10.1186/s12913-022-08238-1

**Published:** 2022-07-08

**Authors:** Matteo Vergani, Fethi Mansouri, Enqi Weng, Praveena Rajkobal

**Affiliations:** Alfred Deakin Institute for Citizenship and Globalisation, Deakin University, Melbourne, Australia

**Keywords:** Ethnic, Multicultural, Ethno-specific, Migrant, CALD

## Abstract

**Background:**

People of culturally and linguistically diverse (CALD) background face significant barriers in accessing effective health services in multicultural countries such as the United States, Canada, Europe and Australia. To address these barriers, government and nongovernment organisations globally have taken the approach of creating ethno-specific services, which cater to the specific needs of CALD clients. These services are often complementary to mainstream services, which cater to the general population including CALD communities.

**Methods:**

This systematic review uses the Evidence Gap Map (EGM) approach to map the available evidence on the effectiveness of ethno-specific and mainstream services in the Australian context. We reviewed Scopus, Web of Science and PubMed databases for articles published from 1996 to 2021 that assessed the impact of health services for Australian CALD communities. Two independent reviewers extracted and coded all the documents, and discussed discrepancies until reaching a 100% agreement. The main inclusion criteria were: 1) time (published after 1996); 2) geography (data collected in Australia); 3) document type (presents results of empirical research in a peer-reviewed outlet); 4) scope (assesses the effectiveness of a health service on CALD communities). We identified 97 articles relevant for review.

**Results:**

Ninety-six percent of ethno-specific services (i.e. specifically targeting CALD groups) were effective in achieving their aims across various outcomes. Eighteen percent of mainstream services (i.e. targeting the general population) were effective for CALD communities. When disaggregating our sample by outcomes (i.e. access, satisfaction with the service, health and literacy), we found that 50 % of studies looking at mainstream services’ impact on CALD communities found that they were effective in achieving health outcomes. The use of sub-optimal methodologies that increase the risk of biased findings is widespread in the research field that we mapped.

**Conclusions:**

Our findings provide partial support to the claims of advocacy stakeholders that mainstream services have limitations in the provision of effective health services for CALD communities. Although focusing on the Australian case study, this review highlights an under-researched policy area, proposes a viable methodology to conduct further research on this topic, and points to the need to disaggregate the data by outcome (i.e. access, satisfaction with the service, health and literacy) when assessing the comparative effectiveness of ethno-specific and mainstream services for multicultural communities.

**Supplementary Information:**

The online version contains supplementary material available at 10.1186/s12913-022-08238-1.

## Background

Delivering effective health services for culturally and linguistically diverse (CALD) communities in multicultural societies raises important policy challenges pertaining to the need to support diversity and multiculturalism whilst uplifting the principles of national citizenship and social inclusion [1]. On the one hand, government and non-government organisations globally have highlighted the critical role of targeted services for the support and empowerment of vulnerable groups in order to support their social integration [2–6]. These services – often referred to as ethno-specific services – aim to tackle the barriers that people from CALD backgrounds face when accessing mainstream services, especially in relation to language [7–9], lack of trust [10], and stigma [11, 12], among others. On the other hand, governments such as Germany, Sweden, and Denmark have focused on developing civic integration strategies through the mainstreaming of services catering to all demographics regardless of ethnic or religious backgrounds [13].

CALD communities are inherently heterogeneous and often include different generations of migrants, asylum seekers and refugees. Each of these groups has different healthcare needs based on their English language proficiency, age, religious affiliation, socio-economic status, cultural backgrounds and period of settlement [14]. Because of the complex, internal diversity of these communities, the health needs of their individual members can also be highly divergent [15]. Previous research suggests that being able to identify patients based on their cultural backgrounds can improve healthcare professionals’ provision of care, including preventative care [14].

An illustrative example is dementia care for CALD communities: research has found that refugees tend to have a higher chance of developing dementia compared to the general population [16, 17]. However, CALD patients tend to have an cultural view of dementia as a natural part of the aging process [16], which can impede early intervention [18]. Additionally, for CALD patients with dementia, culturally appropriate services are critical since they tend to lose their ability to speak a second language and revert back to their first language [17]. However, previous studies found that there is a lack of culturally appropriate healthcare provision for refugees [19]. This example highlights the importance of ethno-specific services for CALD communities, and particularly for vulnerable groups like refugees, who experience numerous barriers to access and receive quality health services from mainstream providers.

### Aim

Using Australia as a case study, this article aims to provide a systematic mapping of the effectiveness of mainstream and ethno-specific health services targeting CALD groups. To achieve this aim, we will use the Evidence Gap Map (EGM) approach, which is broader in scope than systematic reviews because it aims to map a range of heterogeneous interventions and outcomes across various health sub-fields, as opposed to systematic reviews that aim to synthetise evidence and usually focus on a more homogeneous field and set of interventions and/or outcomes [20].

### Population, phenomenon of interest, context and outcomes

Our population of interest are CALD groups in Australia. We operationalised the term CALD as referring to people: from a non-English speaking country of birth, who speak languages other than English at home, have low English proficiency, who identify with a non-Anglo-Saxon/Celtic culture, who self-identify as a migrant, and those who are asylum seekers and refugees. All included studies utilise at least one of these operationalisations to identify their participants. Aboriginal Australians (that include Torres Strait Islanders) are not included in this category; they deserve a separate recognition in the Australian context [21].

Our phenomenon of interest are health services for CALD communities. Consistently with the EGM approach, we adopted broad definitions of what we envision as a health service [22]. We conceptualised *services* operationally as including any strategy, technique, approach, activity, campaign, training, program, directive, or funding/organisational change for the benefit of CALD communities. Within this approach, a *health* outcome includes both physical and mental health domains, and can be measured through self-reported instruments, observations or any other qualitative or quantitative instrument. Examples of health services that we included in our review are community education programs to raise awareness about sexual health or oral health, immunisation campaigns in targeted migrant communities or interventions to improve refugees’ mental health.

Australia is an ideal context to investigate the provision of ethno-specific and mainstream health services to diverse communities for three key reasons. Firstly, the provision of ethno-specific services has a long history in Australia. Indeed, when the White Australia Policy was abolished and replaced with multicultural policies from the early 1970s, a service provision infrastructure that factors in service delivery to people from CALD backgrounds was one of the key recommendations of the Galbally Report [23]. This led to the establishment of numerous ethno-specific services. Secondly, since the election of the Conservative government of John Howard in 1996, Australia experienced a policy shift towards the mainstreaming of services to CALD communities, with the introduction of policies that would gradually reduce or in many cases abolish certain social security benefits and welfare services for new immigrants and shift the balance of the dual system of mainstream and ethno-specific services provision for CALD communities in Australia [24, 25]. Thirdly, Australia has a distinctive and strong multicultural social fabric, with more than 300 languages spoken in Australian homes, more than 100 religions and more than 300 different ancestries recorded by the latest 2016 Census [26]. As of 30 June 2018, 29% of Australia’s resident population was born overseas [27], with more than one in five Australians (21%) speaking a language other than English at home [26].

In terms of outcomes, we considered a wide range of health-related variables, including hospitalisation rates (and other indicators of hospital performance), knowledge of and attitudes towards diseases/treatments (as long as they are the outcome of service provision), biomedical outcomes (blood glucose levels, BMI), among others. We grouped the outcome variables into four types: the satisfaction with the service (which in our studies was measured either as perceived by the clients or by the service providers); the service accessibility for CALD communities (i.e. utilisation of the service); the health outcomes for the CALD clients; the education outcomes (e.g. knowledge scores related to health education).

## Methods

### Literature search and study selection

In February 2021, we conducted searches in Scopus, Web of Science and PubMed using the search strings reported in Table 1. We were specific in selecting these three databases as they offer the most synthetic, cost-effective, comprehensive and complementary coverage of literature in the domains of health and social sciences, especially those published after 1996 [28–30]. We limited our search to peer-reviewed academic research published after 1996, when the then Conservative government led by former PM John Howard introduced significant changes to Australia’s migration and multicultural policies, which gradually shifted the service provision models from an ethno-specific approach towards a mainstreaming of services to CALD communities [24, 25]. We included keywords associated with service domains other than health, such as employment and housing, so that we were able to capture services that address secondary health outcomes for CALD communities, such as mental health issues arising out of unemployment and homelessness [31]. In order to capture documents focusing on services for CALD communities, we used terms that reflect how the literature conceptualised and measured being CALD, i.e. “CALD”, “migrant”, “refugee”, “asylum seeker”.

**Table 1:** Search strings used for the documents search

Date	Database	Search string	Number of hits
13/02/21	Scopus	TITLE-ABS-KEY (evaluat* OR assess* OR impact) AND TITLE-ABS-KEY (service* OR program* OR initiativ* OR scheme* OR plan* OR strateg* OR polic* OR settlement) AND TITLE-ABS-KEY (health OR hous* OR employ* OR homeless* OR job) AND TITLE-ABS-KEY (“CALD” OR *migrant OR refugee* OR “asylum seeker*”) AND TITLE-ABS-KEY (“Australia”)	789
13/02/21	Web of Science	TS = (evaluat* OR assess* OR impact) AND TS = (service* OR program* OR initiativ* OR scheme* OR plan* OR strateg* OR polic* OR settlement) AND TS = (health OR hous* OR employ* OR homeless* OR job) AND TS = (“CALD” OR *migrant OR refugee* OR “asylum seeker*”) AND TS = Australia	757
13/02/21	PubMed	(evaluat* OR assess* OR impact) AND (service* OR program* OR initiativ* OR scheme* OR plan* OR strateg* OR polic* OR settlement) AND (health OR hous* OR employ* OR homeless* OR job) AND (“CALD” OR migrant OR refugee* OR “asylum seeker*”) AND Australia	774

**Fig. 1:**
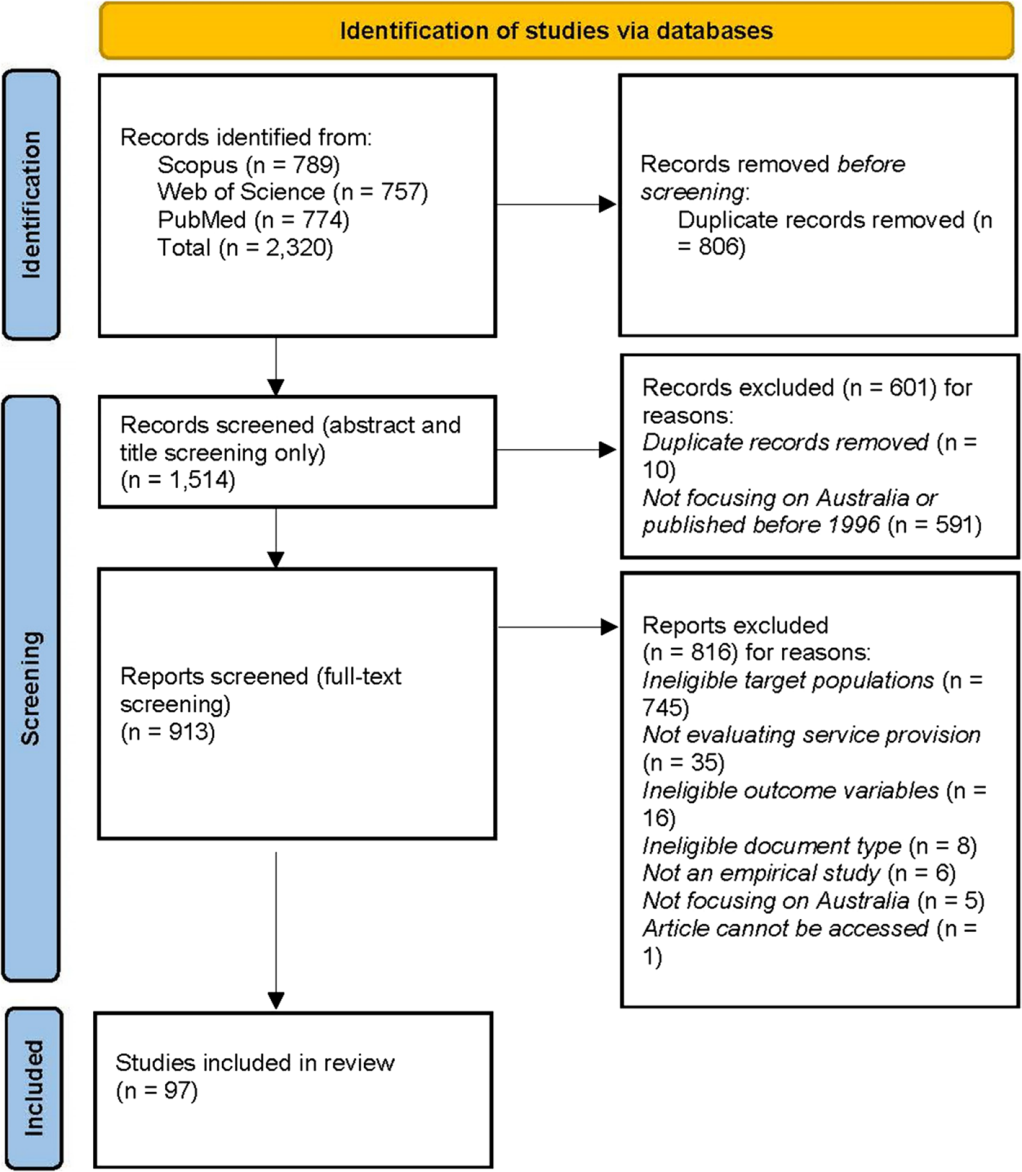
PRISMA flow chart

The three searches outlined in Table 1 retrieved a total of 2320 documents. We removed duplicates and performed title and abstract screening based on: 1) time (published between January 1996 and February 2021); and 2) geography (data collected in Australia). At the end of this process, we obtained 913 studies eligible for full-text screening. Two researchers conducted the full-text screening using the following six criteria: 1) time (published after 1996); 2) geography (data collected in Australia); 3) document type (presents results of empirical research in a peer-reviewed outlet); 4) scope (assesses the effectiveness of a health service on CALD communities); 5) ineligible document type (letters, book reviews and notes to editors were excluded); and 6) access (if we could not access the article via our library databases, the article was excluded). We minimised access issues as much as possible by conducting thorough online searches and by using all subscriptions available to our university library to locate the documents. All coding discrepancies were discussed during team meetings. All types of study designs were included. A sample of 97 documents were eligible for coding and analysis. Figure 1 reports the PRISMA flow diagram of our review process.

### Critical appraisal

We used the Critical Appraisal Skill Programme (CASP) tool to assess the quality of the included studies [32]. We selected the CASP tool because it is well-established in the literature and it offers tailored checklists for a range of qualitative and quantitative study designs, which reflects the diversity of designs in our sample of documents. In line with Cochrane guidelines, we avoided using the tool to apply scores to calculate the total quality score of each article because not all domains of quality are equal, and because our review includes a diverse set of designs and methodologies that are not directly comparable [33]. Instead, we assigned studies to one of three bands: higher quality, medium quality, and lower quality as suggested by Long et al. [32] We assigned the band “higher quality” when the study complied with all the optimal quality requirements in the CASP checklist. We assigned the band “lower quality” when the study had major flaws (e.g. the study aims were unclear, the methods were not appropriate to address the aims or the sample was not recruited in an acceptable way in relation to the aims), which the review team decided as being the tipping point criteria based on this review aims and context. All other documents (i.e. the ones that did not comply with all the optimal quality requirements of the CASP checklist but did not have major flows) were categorised as being in the “medium quality” band.

### Data extraction

Following White et al.’s [20] guidelines for conducting EGMs, we adopted an inductive approach to create sub-categories of health services, such as maternity and child services, sexual health, oral health (see Table 2 for the full list). As our review aims to map the effectiveness of ethno-specific and mainstream health services, we created a binary variable capturing whether the study was effective or not for CALD communities. The effectiveness was determined by two coders, based on whether the service’s outcomes (as measured in the article) were achieved. The criteria for classifying included papers as Yes in our study for effectiveness were different between qualitative and quantitative studies. For the quantitative studies, we looked at whether included papers reported statistically significant findings for the intervention/program for the CALD group. For the qualitative studies, we looked at whether included papers reported qualitative evidence that the intervention/program achieved its purposed outcomes. For example, if a study found that CALD communities faced significant barriers to access a certain health service (see for example Mahimbo et al [34]), we coded the service as being “not effective”. If a study found that a certain health service was able to improve access for CALD communities (see for example Smith and Gallego [35]), we coded it as “effective”. In Annex 1, we provide the full list of the 97 studies included in our review, with a narrative description of the findings and the effectiveness criterion for each article. Two researchers coded the documents. Discrepancies were discussed by the researchers and resolved through consensus.

**Table 2:** Coding scheme

*Variables*	*Values*
Types of health service	1 = Generic health services (e.g. general practitioners, hospitals) 2 = Elderly health care 3 = Immunisation and prevention of communicable diseases (e.g. malaria, tuberculosis) 4 = maternity and child services 5 = prevention of obesity, diabetes and cardio-vascular diseases 6 = cancer and palliative care 7 = mental health 8 = sexual health 9 = oral health 10 = other
Service target population	1 = the service targets a CALD group 2 = the service targets the general population
Index of ethno-specificity of the service^a^	1 = presence of translation or interpreter services 0 = absence of translation or interpreter services
	1 = presence of CALD staff delivering the service 0 = absence of CALD staff delivering the service
	1 = delivery of ethno-specific and cultural training to staff 0 = absence of ethno-specific and cultural training to staff
	1 = tailoring of service content to the CALD target group 0 = absence of service content to the CALD target group
Stage of service implementation	1 = pilot program 2 = intervention 3 = ongoing service
Outcome type	1 = satisfaction with the service 2 = access to the service 3 = health outcomes 4 = education outcomes
Sample quality index^b^	1 = presence of a sample of CALD clients 0 = absence of a sample of CALD clients
	1 = presence of a control or comparison group of non-CALD clients 0 = absence of a control or comparison group of non-CALD clients
Methodology	1 = qualitative 2 = quantitative 3 = mixed methods
Effectiveness of the service (see Appendix 1)	1 = effective 0 = not effective

^a^The index ranges from 0 to 4, with one point attributed for each ethno-specific feature of the service. ^b^The index has three levels: lower sample quality (for articles having no CALD clients in the sample), medium sample quality (for articles having at least one CALD client in the sample), and higher sample quality (for articles including both CALD clients and a control or comparison group of non-CALD clients)

To differentiate between mainstream and ethno-specific services, we adopted two parallel approaches. Firstly, we looked at whether the service exclusively targeted a CALD group or the service targeted the general population (and the study reported on its effectiveness for a CALD group). Secondly, we created an index to capture the ethno-specific features of the services. The index was created by attributing 1 point to each of the following four characteristics: 1) the presence of translators or interpreters; 2) the presence of CALD staff delivering the service; 3) the provision of ethno-specific and cultural training to the staff, and 4) the tailoring of the service content to the CALD target group. We used these two parallel and nuanced approaches because we conceptualise ethno-specific and mainstream services as poles of a continuum, rather than as rigid and binary categories [18]. In both academia and health practice, there has been a general reductionist, binary view of services for CALD communities as either mainstream or ethno-specific, where mainstream services cater to a general population and ethno-specific services cater to CALD communities [5, 18, 36–38]. In reality, mainstream service providers may at times adopt ethno-specific strategies, such as the provision of translated materials or interpreters, the hire of CALD staff, the provision of cultural responsive training to their staff and the customisation of their services to factor CALD communities [18].

We coded whether the studies employed a qualitative, quantitative or mixed methodology to describe the diversity of approaches and study designs in our sample. We also looked at whether each article was assessing a service at different stages of implementation, which could be a pilot program (that is, a one-off service implemented by a service provider), an intervention (that is, a one-off service implemented by a university research team for the purpose of testing its efficacy), or an ongoing service (that is, a service implemented by a service provider on an ongoing basis). Finally, we coded whether or not the article considered the service as effective for CALD clients. Table 2 reports our full coding scheme.

### Analytical approach

We used SPSS to perform univariate and bivariate statistical analyses, and used Chi-Square tests to examine whether there were statistically significant differences in the proportions of different types of services and their outcomes.

## Results

### Descriptive data

In the 97 articles included, 53 (54.6%) of the services reviewed specifically targeted a CALD group, and 44 (45.4%) targeted the general population. In terms of Index of ethno-specificity of the service, 29 (29.9%) used translations or interpreters, 20 (20.6%) used CALD staff to implement the service, 29 (29.9%) adapted the content to the target communities’ cultures, and 11 (11.3%) provided culturally specific training to the staff delivering the service (see Table 2). In terms of stage of implementation of the service, 16 (16.5%) were pilot programs, 28 (18.9%) were interventions, and 53 (54.6%) were ongoing services. When looking at the outcome measures considered in the articles, 29 (29.9%) focused on the quality of satisfaction with the service, 25 (25.8%) on the access to the service, 31 (32%) on clients’ health outcomes, and 12 (12.4%) on education outcomes.

Thirty-three studies (34%) used a qualitative, 42 (43.3%) a qualitative, and 22 (22.7%) a mixed methodology, and 59 studies (60.8%) found the service to be effective and 38 (39.2%) not effective for CALD clients. As for the types of health services assessed in the articles included in the review, 25 focused on mental health services, 15 on General Practitioners and Hospitals, 12 on maternity and child care services, 11 on oral health, 11 on the prevention of obesity, diabetes and cardiovascular disease, 7 on immunisation and prevention of communicable diseases, 6 on cancer and palliative care, and the remaining 8 on other categories (e.g. sexual health, elderly health care).

Forty-four percent of the studies looking at the satisfaction with the service and 52 % of the studies looking at access to the service use qualitative methodologies only. Conversely, the majority of the studies looking at health outcomes (61.3%) and education outcomes (58.3%) use quantitative methodologies only. Thirty-two studies (33%) were found to be of higher quality, 30 (31%) of lower quality, and 35 (36%) of medium quality. The studies with higher sample quality are 24.1% of the studies looking at service quality, 48% of studies looking at access to service, 32.3% of studies looking at health outcomes and 25% of studies looking at health outcomes. The studies with lower sample quality are 31% of the studies looking at service quality, 16% of studies looking at access to service, 41.9% of studies looking at health outcomes and 33.3% of studies looking at health outcomes.

### Main results

When looking at the effectiveness of ethno-specific and mainstream services across all health outcomes, we found that 51 (96.2%) services specifically targeting CALD groups were effective in achieving their aims across various outcomes. Conversely, 36 (81.8%) services targeting the general population were not effective. The differences between groups were statistically significant, X^2^ (1, 97) = 61.45, *p* < .001. Subsequently, we disaggregated our sample by different outcomes (i.e. access, satisfaction with the service, health and literacy). Table 3 shows that 17 (94.4%) services targeting CALD communities – but just 1 (10%) services targeting mainstream clients – were effective in terms of clients’ satisfaction. Five services (100%) targeting CALD communities – but only 1 (5%) services targeting mainstream clients – were effective in terms of access to the service. Importantly, 20 (95.2%) services targeting CALD communities were effective in achieving health outcomes, but also 5 (50%) services targeting mainstream clients were effective. Finally, 9 (100%) services targeting CALD communities – and 1 (33.3%) service targeting mainstream clients – were effective in achieving literacy outcomes.

**Table 3:** Evidence gap map framework: service’s target population by types of outcomes and effectiveness

	Service targeting CALD clients	Service targeting mainstream clients	Total
Satisfaction with the service	Eff = 17 Not eff = 1	Eff = 1 Not eff = 10	Eff = 18 Not eff = 11
Access to service	Eff = 5 Not eff = 0	Eff = 1 Not eff = 19	Eff = 6 Not eff = 19
Health outcomes	Eff = 20 Not eff = 1	Eff = 5 Not eff = 5	Eff = 25 Not eff = 6
Literacy outcomes	Eff = 9 Not eff = 0	Eff = 1 Not eff = 2	Eff = 10 Not eff = 2
Total	Eff = 51 Not eff = 2	Eff = 8 Not eff = 36	Eff = 59 Not eff = 38

*Eff* Effective, *Not eff* Not effective

To test for the robustness of our findings, we re-conducted the analyses by operationalising ethno-specific services as having at least one ethno-specific feature (i.e. translation or interpreters, CALD staff delivering the service, cultural training to non-CALD staff, content adapted to CALD group’s culture), and mainstream services as having no ethno-specific features. We found that 41 (95.3%) of services having at least one ethno-specific feature were effective, and 36 (66.7%) of services having no ethno-specific features were not effective for CALD groups. The differences between groups were statistically significant, X^2^ (3, 97) = 38.88, *p* < .001. Table 4 reports the results of the analyses disaggregating by outcome. Only 3 services (23%) with no ethno-specific features were effective in achieving CALD clients’ satisfaction with the service, and only 2 (9.5%) in achieving effective access to the service for CALD clients. However, 10 services (66.7%) with no ethno-specific features were effective in achieving CALD clients’ health outcomes, and 3 (60%) in achieving effective literacy outcomes.

**Table 4:** Evidence gap map framework: service’s ethno-specific features by types of outcomes and effectiveness

	Service with one or more ethno-specific features	Service with no ethno-specific features	Total
Satisfaction with the service	Eff = 15 Not eff = 1	Eff = 3 Not eff = 10	Eff = 18 Not eff = 11
Access to service	Eff = 4 Not eff = 0	Eff = 2 Not eff = 19	Eff = 6 Not eff = 19
Health outcomes	Eff = 15 Not eff = 1	Eff = 10 Not eff = 5	Eff = 25 Not eff = 6
Literacy outcomes	Eff = 7 Not eff = 0	Eff = 3 Not eff = 2	Eff = 10 Not eff = 2
Total	Eff = 41 Not eff = 2	Eff = 18 Not eff = 36	Eff = 59 Not eff = 38

*Eff* Effective, *Not eff* Not effective

## Discussion

This article mapped existing and available research evidence on the effectiveness of ethno-specific and mainstream health services for CALD communities in Australia. To strengthen the robustness of our findings, we operationalised ethno-specific services in two ways: the first as services targeting CALD communities, and the second as services having a range of ethno-specific features. In both operationalisations, the findings are consistent. Across all outcomes, we find that ethno-specific services are significantly more likely to be found effective for CALD communities than mainstream services. However, when disaggregating the sample by different outcome measures, we find that mainstream services are found more effective for CALD clients in achieving health and literacy outcomes than access and satisfaction with the service - although they remain comparatively less effective than ethno-specific services across all outcomes.

Our study identifies significant gaps in knowledge in the field of research mapped in our EGM. As the studies on the effectiveness of health services for CALD clients are fragmented across numerous health subfields, there is insufficient data to test whether there are significant differences within each subfield, or whether some variables might moderate the effect of a type of service on a certain outcome variable. More evidence is needed for such testing. Our appraisal of the available research evidence suggests that there is a widespread use of sub-optimal methodologies with about one third of the documents (30%) having lower quality. Finally, it is important to consider that 45.4% (*N* = 44) of the evidence that we mapped in this article consists of studies that assessed the effectiveness of pilot programs (*N* = 16) and one-off interventions (*N* = 28). This means that our EGM does not provide an overview of the services that currently exist, but include programs that were piloted or trialled but never implemented.

Mindful of the potential distortion and confirmation bias in the empirical studies that we reviewed, and mindful that almost half of the articles included in our review assessed one-off and pilot interventions that might be different from established services, we interpret our findings as providing partial support to the claims of advocacy stakeholders that mainstream services have limitations in the provision of effective health services for CALD communities. This gap in the provision of ethno-specific services would place already vulnerable communities at further risk of social marginalisation and exclusion with serious human rights implications [4, 36, 37, 39, 40].

## Conclusion

Our study highlights the well documented psychosocial challenges related to settlement, civic participation and integration for CALD communities [41–45], as well as the need that appropriate appraisals of services are conducted, focusing on overall integration experiences, social cohesion, human rights and citizenship. Our approach to understand service provision for CALD communities reflects T.H. Marshall’s theory of citizenship [46], which sees social rights as the cornerstone for citizen’s proactive civic and political engagement. And within the social dimension of citizenship, service provision is a key platform for enacting core citizenship rights. The dearth of research into this important area of public policy limits the efforts of policy-makers and service providers in designing, planning and administering high-quality services for increasingly diverse communities. On a more long-term basis, understanding the effectiveness of different types of mainstream and ethno-specific approaches to health service delivery is of key importance for several reasons.

First, immigrant populations are becoming more heterogeneous and societies are becoming more ethno-religiously diverse overall, as characterised by Vertovec [47] in terms of ‘super-diversity’. Second, the number of native-born people with immigrant parents is also growing, resulting in a rising number of second- and third-generation migrants whose needs are different from those of both first-generation and recently settled migrants. Third, in the current social and political climate, there is a growing concern that “multiculturalism has failed”, and that some communities are becoming more segregated from mainstream society than others and thus pose a potential threat to social cohesion [48, 49]. Fourth, in a period of economic downturn, governments often pursue social policies that are supposed to be cost-effective whilst remaining responsive to society as a whole without appearing to prioritise certain groups over others [3]. Evidence for policymakers on the effectiveness of ethno-specific services for CALD communities is especially needed during a health crisis like the COVID-19 pandemic [50].

## Supplementary Information

**Additional file 1.**

## Data Availability

All data generated or analysed during this study are included in this published article and its supplementary information files.

## Abbreviations

CALD: Culturally And Linguistically Diverse
EGM: Evidence Gap Maps
