# Supplementary material for: The effectiveness of ethno-specific and mainstream health services: an evidence gap map
Source: BMC Health Serv Res. 2022 Jul 8;22:879. doi: 10.1186/s12913-022-08238-1 (PMC9263048; doi:10.1186/s12913-022-08238-1)
Supplement: Supplementary file 1 — Additional file 1. [file 12913_2022_8238_MOESM1_ESM.docx]

**ANNEX 1.**

| **Reference** | **Aim** | **Effectiveness (narrative)** | **Effectiveness (Y/N)** | **Critical appraisal (Higher, medium, lower quality)** |
| --- | --- | --- | --- | --- |
| Abdi, I., Menzies, R., & Seale, H. (2019). Barriers and facilitators of immunisation in refugees and migrants in Australia: an east-African case study. *Vaccine, 37*(44), 6724-6729. doi:https://doi.org/10.1016/j.vaccine.2019.09.025 | To assess access to immunisation services in East African communities in New South Wales and Victoria. | The study participants experienced significant barriers in accessing the service. The main barriers reducing the effectiveness of immunisation in east African communities were language barriers, low risk perception, and a lack of education while the possible enablers were the availability of resources in the participants’ language, a reminder system, and the involvement of community organisations. | N | Medium |
| Agu, J., Lobo, R., Crawford, G., & Chigwada, B. (2016). Migrant Sexual Health Help-Seeking and Experiences of Stigmatization and Discrimination in Perth, Western Australia: Exploring Barriers and Enablers. *Int. J. Environ. Res. Public Health, 13*(5), 485. doi:https://doi.org/10.3390/ijerph13050485 | To assess access to sexual health services among sub-Saharan African, Southeast Asian, and East Asian migrants | The study participants experienced significant barriers in accessing the service. Common barriers to accessing sexual health services were sociocultural and religious influence, and financial constraints while the factors increasing the effectiveness of health services were identified to be awareness, recognition of cultural differences, and reducing stigma and discrimination. | N | Higher |
| Alizadeh, S., & Chavan, M. (2020). Perceived Cultural Distance in Healthcare in Immigrant Intercultural Medical Encounters. *International Migration, 58*(4), 231-254. doi:https://doi.org/10.1111/imig.12680 | To assess healthcare experiences among immigrants. | Cultural distance had a significant negative impact on the perceived quality of outpatient care while cultural competence was identified to be a factor that enhanced the effectiveness of outpatient care. | N | Lower |
| Aminisani, N., Armstrong, B. K., & Canfell, K. (2012). Cervical cancer screening in Middle Eastern and Asian migrants to Australia: A record linkage study. *Cancer Epidemiology, 36*(6), e394-e400. doi:https://doi.org/10.1016/j.canep.2012.08.009 | To assess cervical screening behaviour among migrants | The program was more effective in attracting response from Australian-born women than women from Asian and Middle-eastern backgrounds. | N | Higher |
| Arora, A., Al-Salti, I., Murad, H., Tran, Q., Itaoui, R., Bhole, S., . . . Manohar, N. (2018). Adaptation of child oral health education leaflets for Arabic migrants in Australia: a qualitative study. *BMC Oral Health, 18*(10), 1-10. doi:DOI 10.1186/s12903-017-0469-z | To gauge Arabic-speaking mothers’ views on the effectiveness of the existing oral health education leaflets for young children and the tailored versions of the same leaflets. | Tailored oral health education leaflets were more effective among Arabic-speaking migrants. | N | Higher |
| Arora, A., Maharaj, R., Naidu, S., Chimoriya, R., Bhole, S., Nash, S., & Jones, C. (2021). Views of Indian Migrants on Adaptation of Child Oral Health Leaflets: A Qualitative Study. *Children, 8*(1), 1-13. doi:https://doi.org/10.3390/children8010028 | To gauge Hindi-speaking mothers’ views on the effectiveness of the existing English language oral health education leaflets and the translated Hindi versions of the same leaflets. | The Hindi translation of the standard English version of oral health leaflets was less effective among the Hindi-speaking mothers than the simplified English version of the standard English-language leaflet. | N | Higher |
| Baker, F., & Jones, C. (2006). The effect of music therapy services on classroom behaviours of newly arrived refugee students in Australia—a pilot study. *Emotional and Behavioural Difficulties, 11*(4), 249-260. doi:10.1080/13632750601022170 | To examine the impact of a short-term music therapy program on the classroom behaviour of newly arrived refugee students in a secondary school for ‘English as a Second Language’. | The music therapy program was effective in reducing hyperactivity and aggression in students. | Y | Lower |
| Barrett, P. M., Moore, A. F., & Sonderegger, R. (2000). The FRIENDS Program for Young Former-Yugoslavian Refugees in Australia: A Pilot Study. *Behaviour Change, 17*(3), 124-133. doi:10.1375/bech.17.3.124 | To investigate the effectiveness of the FRIENDS program in reducing anxiety among former- Yugoslavian teenage refugees. | The FRIENDS program was effective in reducing internalising symptoms among the participating group of the former- Yugoslavian teenage refugees. | Y | Medium |
| Barrett, P. M., Sonderegger, R., & Sonderegger, N. L. (2001). Evaluation of an Anxiety-prevention and Positive-coping Program (FRIENDS) for Children and Adolescents of Non-English-speaking Background. *Behaviour Change, 18*(2), 78-91. doi:10.1375/bech.18.2.78 | To investigate the effectiveness of the FRIENDS program in reducing anxiety among culturally diverse migrant groups in Australia. | The FRIENDS program was effective in reducing anxiety among the participating group of CALD migrants in comparison to the wait-listed participants. | Y | Medium |
| Barrett, P. M., Sonderegger, R., & Xenos, S. (2003). Using Friends to Combat Anxiety and Adjustment Problems among Young Migrants to Australia: A National Trial. *Clinical Child Psychology and Psychiatry, 8*(2), 241-260. doi:10.1177/1359104503008002008 | To investigate the effectiveness of the FRIENDS program in reducing psychological distress among young migrants in Australia from non-English speaking background (NESB). | The FRIENDS program was effective in reducing psychological distress among the participating group of young migrants in comparison to the wait-listed participants. | Y | Medium |
| Beauchamp, A., Mohebbi, M., Cooper, A., Pridmore, V., Livingston, P., Scanlon, M., . . . Osborne, R. (2020). The impact of translated reminder letters and phone calls on mammography screening booking rates: Two randomised controlled trials. *PLoS ONE, 15*(1), 1-16. doi:https://doi.org/10.1371/journal.pone.0226610 | To evaluate how effective translated screening routine reminder letters or phone calls are in Arabic and Italian women’s decision to screen. | Telephone call reminders proved to be the most effective method in contributing to women’s decision to screen while translated reminder letters also an influential method. | Y | Higher |
| Biro, M. A., & East, C. (2017). Poorer detection rates of severe fetal growth restriction in women of likely refugee background: A case for re-focusing pregnancy care. *Australian & New Zealand Journal of Obstetrics & Gynaecology, 57*(2), 186-192. doi: https://doi.org/10.1111/ajo.12593 | To examine the impact of likely refugee background on severe fetal growth restriction (FGR) in a singleton pregnancy undelivered by 40 weeks. | The likelihood of mothers of refugee background giving birth to a severely growth-restricted baby after 40 weeks was at two and half times the odds compared to mothers of nonrefugee background indicating that Victorian Perinatal Services Performance was less effective for mothers of refugee background. | N | Higher |
| Blignault, I., Woodland, L., Ponzio, V., Ristevski, D., & Kirov, S. (2009). Using a multifaceted community intervention to reduce stigma about mental illness in an Australian Macedonian community. *Health promotion journal of Australia, 20*(3), 227-233. doi:https://doi.org/10.1071/HE09227 | To assess a community intervention to reduce the stigma of mental illness programs among the Macedonian community in south-east Sydney. | The intervention designed for the Macedonian community was effective in making a positive change in their attitudes and beliefs towards mental illness. | Y | Medium |
| Block, K., Cross, S., Riggs, E., & Gibbs, L. (2014). Supporting schools to create an inclusive environment for refugee students. *International Journal of Inclusive Education, 18*(12), 1337-1355. doi:10.1080/13603116.2014.899636 | To present the results of an evaluation of the School Support Programme implemented in schools in Victoria, Australia for refugee-background students. | The School Support Programme was an effective model in providing inclusive education for students from refugee-backgrounds. | Y | Medium |
| Bond, L., Giddens, A., Cosentino, A., Cook, M., Hoban, P., Haynes, A., . . . Glover, S. (2007). Changing cultures: enhancing mental health and wellbeing of refugee young people through education and training. *Promot Educ, 14*(3), 143-149. doi:https://pubmed.ncbi.nlm.nih.gov/18154223/ | To assess the Changing Cultures Project, a project which examined models of appropriate and accessible education and training for improving mental health for refugee and newly arrived young people. | Changing Cultures Project was effective in delivering positive outcomes for refugee young people through speaking to the issues of program development and delivery, organisational development and capacity building, and community development and evaluation. | Y | Medium |
| Bradley, G. M., Couchman, G. M., Perlesz, A., Nguyen, A. T., Singh, B., & Riess, C. (2006). Multiple-family group treatment for English- and Vietnamese-speaking families living with schizophrenia. *Psychiatr Serv, 57*(4), 521-530. doi:10.1176/ps.2006.57.4.521 | To explore the effectiveness of multiple-family group treatment for a newly arrived non-English speaking migrant group, first-generation Vietnamese families, and for English-speaking families. | Multiple-family group treatment was found to be an effective cognitive-behavioural intervention in the treatment of schizophrenia for non-English speaking migrant populations. | Y | Higher |
| Broadbent, R., Cacciattolo, M., & Carpenter, C. (2007). A Tale of Two Communities: Refugee Relocation in Australia. *Australian Journal of Social Issues, 42*(4), 581-601. doi:https://doi.org/10.1002/j.1839-4655.2007.tb00079.x | To assess the impact of a refugee relocation project as a labour and population renewal strategy. | In order to be effective, the refugee relocation projects as a labour and population renewal strategy should go beyond community acceptance of such strategies to address issues and challenges persistent in the policy arena regarding mental health, employment and housing. | Y | Lower |
| Brophy-Williams, S., Boylen, S., Gill, F. J., Wilson, S., & Cherian, S. (2020). Use of professional interpreters for children and families with limited English proficiency: The intersection with quality and safety. *Journal of Paediatrics and Child Health, 56*(8), 1201-1209. doi:10.1111/jpc.14880 | To assess the hospital-wide use of interpreters for low English proficiency in a tertiary hospital across emergency (ED), outpatient and inpatient services. | The use of professional interpreters for patients with low English proficiency was inadequate, impacting on the health outcomes for and compliance in patients with low English proficiency. | N | Medium |
| Brown W.J.; Lee C.; Oyomopito R. (1996). Effectiveness of a bilingual heart health program for Greek-Australian women. *Health Promotion International, 11*(2), 117-125. doi:https://academic.oup.com/heapro/article/11/2/117/634816 | To examine the impact of a 12-week minimal-intervention heart health program on a community sample of Greek-Australian women. | The intervention was successful, demonstrating that interventions specifically tailored for women from non-English-speaking backgrounds (NESB) are more effective in modifying cardiovascular risk factors as they eliminated sociocultural and linguistic barriers to participation. | Y | Lower |
| Bruce, D. G., Davis, W. A., Cull, C. A., & Davis, T. M. E. (2003). Diabetes education and knowledge in patients with type 2 diabetes from the community: The Fremantle Diabetes Study. *Journal of Diabetes and its Complications, 17*(2), 82-89. doi:https://doi.org/10.1016/S1056-8727(02)00191-5 | To assess the impact of diabetes education programs on the knowledge of a cohort of patients with type 2 diabetes. | Education programs, visits to dieticians, and engaging in self-monitoring of blood glucose (SMBG) were effective methods in improving diabetes knowledge in participating patients in comparison to those who were older, had limited schooling, who were low in English proficiency and/or from Southern European or indigenous Australian ethnic groups. | N | Lower |
| Caperchione, C. M., Kolt, G. S., & Mummery, K. (2013). Examining Physical Activity Service Provision to Culturally and Linguistically Diverse (CALD) Communities in Australia: A Qualitative Evaluation. *PLoS ONE, 8*(4), 1-8. doi:https://doi.org/10.1371/journal.pone.0062777 | To assess the access and quality perception of physical activity services/initiatives for CALD groups. | Most services for CALD groups were not designed considering the expectations and requirements of CALD communities, especially socio-cultural (e.g., gender, language, context of health) and environmental (e.g., transportation) needs, which were identified as the barriers that should be addressed to make future physical and health promotion activities effective in attracting the participation of CALD communities. | N | Higher |
| Cheng, I.-H., McBride, J., Decker, M., Watson, T., Jakubenko, H., & Russo, A. (2019). The Asylum Seeker Integrated Healthcare Pathway: a collaborative approach to improving access to primary health care in South Eastern Melbourne, Victoria, Australia. *Australian Journal of Primary Health, 25*, 6-12. doi:10.1071/PY18028 | To determine whether the Asylum Seeker Integrated Healthcare Pathway improved use of health services among asylum seekers. | The Asylum Seeker Integrated Healthcare Pathway effectively improved the use of health services among newly arrived asylum seekers settling into the South Eastern Region of Melbourne. | Y | Lower |
| Correa-Velez, I., & Ryan, J. (2012). Developing a best practice model of refugee maternity care. *Women and Birth, 25*(1), 13-22. doi:https://doi.org/10.1016/j.wombi.2011.01.002 | To asses maternity care for women from refugee backgrounds. | An effective model of maternity care for women from refugee backgrounds should incorporate the practices of continuity of carer, quality interpreter services, educational strategies for both women and healthcare professionals, and the provision of psychosocial support to women from refugee backgrounds. | N | Medium |
| Cullerton, K., Gallegos, D., Ashley, E., Do, H., Voloschenko, A., Fleming, M., . . . Gould, T. (2016). Cancer screening education: can it change knowledge and attitudes among culturally and linguistically diverse communities in Queensland, Australia? *Health promotion journal of Australia, 27*(2), 140-147. doi:https://doi.org/10.1071/HE15116 | To evaluate the impact of the pilot Cancer Screening Education Program developed by the Ethnic Communities Council of Queensland on knowledge, attitudes, and intentions to participate in screening among culturally and linguistically diverse (CALD) communities living in Brisbane, Queensland. | A culturally customised cancer screening education program was effective in improving knowledge, attitudes about and intentions to participate in cancer screening among CALD communities living in Brisbane, Queensland. | Y | Lower |
| Cyril, S., Green, J., Nicholson, J., Agho, K., & Renzaho, A. (2016). Exploring Service Providers' Perspectives in Improving Childhood Obesity Prevention among CALD Communities in Victoria, Australia. *PLoS ONE, 11*(10), 1-22. doi:doi: 10.1371/journal.pone.0162184 | To assess CALD communities’ access to existing obesity prevention services. | The main factors limiting CALD communities’ participation in the existing obesity prevention services, hindering the effectiveness of these services were low CALD health literacy, lack of knowledge of cultural barriers among service providers, and deficiencies found in the structure and delivery of obesity prevention services. | N | Medium |
| Davidson, N., Skull, S., Calache, H., Chesters, D., & Chalmers, J. (2007). Equitable access to dental care for an at-risk group: a review of services for Australian refugees. *Aust N Z J Public Health, 31*(1), 73-80. doi:https://pubmed.ncbi.nlm.nih.gov/17333613/ | To assess the gaps in service provision and barriers to accessing public dental services for refugees across Australian jurisdictions. | Dental services for refugees were found to be ineffective in rural and regional areas, while barriers to access were identified as the long waiting times, variation in assessment criteria, different eligibility criteria and limited interpreter services. | N | Lower |
| Flynn, M. G., & Brown, L. K. (2015). Treatment of latent tuberculosis in migrants to Victoria. *Communicable diseases intelligence, 39*(4), E578–E583. doi:https://www.agedcare.health.gov.au/internet/main/publishing.nsf/Content/cda-cdi3904-pdf-cnt.htm/$FILE/cdi3904b.pdf | To assess whether the percentage of migrants tested for tuberculosis, offered treatment and completed treatment in Victoria reflect the completion rates shown in other published studies. | The percentage of migrants tested for tuberculosis, offered treatment, and completed treatment in Victoria corroborated the findings from other similar studies. | Y | Higher |
| Gallegos, D., Do, H., To, Q. G., Vo, B., Goris, J., & Alraman, H. (2021). The effectiveness of living well multicultural-lifestyle management program among ethnic populations in Queensland, Australia. *Health promotion journal of Australia, 32*(1), 84-95. doi: https://doi.org/10.1002/hpja.329 | To evaluate the effectiveness of the Living Well Multicultural-Lifestyle Management Program (LWM-LMP) in Queensland, Australia. | The LWM-LMP was effective in improving its participants' lifestyle behaviours and cardiometabolic indicators. | Y | Lower |
| Gartley, T., & Due, C. (2017). The interpreter is not an invisible being: A thematic analysis of the impact of interpreters in mental health service provision with refugee clients. *Australian Psychologist, 52*(1), 31-40. doi:https://doi.org/10.1111/ap.12181 | To examine how the use of interpreters impact on the mental healthcare for refugee clients. | The service provided by interpreters was found to be essential in the provision of mental healthcare for refugee clients. | Y | Higher |
| Gibbs, L., Waters, E., Christian, B., Gold, L., Young, D., de Silva, A., . . . Moore, L. (2015). Teeth Tales: a community-based child oral health promotion trial with migrant families in Australia. *BMJ Open, 5*(6), 1-12. doi:https://bmjopen.bmj.com/content/5/6/e007321 | To assess the impact of The Teeth Tales intervention, a model for child oral health promotion for culturally diverse communities in Australia. | The Teeth Tales intervention was effective in improving oral hygiene and parents’ knowledge of proper tooth brushing methods. | Y | Medium |
| Gould, G., Kerri, V., Michele, G., John, K., & Paul, C. (2010). A multidisciplinary primary healthcare clinic for newly arrived humanitarian entrants in regional NSW: model of service delivery and summary of preliminary findings. *Australian and New Zealand Journal of Public Health, 34*, 326-329. doi:10.1111/j.1753-6405.2010.00535.x | To assess the effectiveness of the Coffs Harbour Refugee Health Clinic during its initial period of operation. | The Coffs Harbour Refugee Health Clinic was effective in providing services to newly arrived humanitarian entrants as the clinic was well utilised by the newly arrived humanitarian entrants in regional New South Wales. | Y | Medium |
| Guajardo, M. G. U., Kelly, C., Bond, K., Thomson, R., & Slewa-Younan, S. (2019). An evaluation of the teen and Youth Mental Health First Aid training with a CALD focus: an uncontrolled pilot study with adolescents and adults in Australia. *International Journal of Mental Health Systems, 13*(73). doi:https://doi.org/10.1186/s13033-019-0329-0 | To evaluate the CALD-focused face-to-face teen (tMHFA) and Youth Mental Health First Aid (YMHFA) training programs. | CALD tMHFA and YMHFA were effective in improving the mental health of CALD youth. | Y | Lower |
| Gunaratnam, P., Sestakova, L., Smith, M., & Torvaldsen, S. (2013). Evaluation of a multilingual oral health DVD for newly arrived refugees. *Health Promotion Journal of Australia 24*(2), 159-159. doi:10.1071/HE13070 | To evaluate the effectiveness of a multilingual DVD providing culturally and linguistically appropriate information to refugees in NSW detailing preventive oral health behaviours. | The multilingual DVD was effective in improving oral health-related knowledge among refugees in NSW. | Y | Lower |
| Hancock, P., Cooper, T., & Bahn, S. (2009). Evaluation of the Integrated Services Pilot Program from Western Australia. *Eval Program Plann, 32*(3), 238-246. doi:10.1016/j.evalprogplan.2008.12.001 | To evaluate the extent to which the Integrated Services Centre (ISC) Pilot Project, a refugee-focused program, met its objectives. | The ISC Pilot Project was effective in meeting the needs mainly of the African refugees, ensuring a timely and holistic service delivery. | Y | Medium |
| Hanes, G., Sung, L., Mutch, R., & Cherian, S. (2017). Adversity and resilience amongst resettling Western Australian paediatric refugees. *Journal of Paediatrics and Child Health, 53*(9), 882-888. doi:https://doi.org/10.1111/jpc.13559 | To conduct an audit of the Princess Margaret Hospital Refugee Health Service (RHS) proformas, health records and initial and 6-month follow-up Strengths and Difficulties Questionnaire (SDQs) for new patients aged 2-16 years between August 2014 and January 2016. | Comprehensive standardised health and psychological screening was identified as an effective method towards identifying target intervention while emphasising the need to further validate the culturally age-appropriate mental health screening tools. | Y | Lower |
| Haralambous, B., Tinney, J., LoGiudice, D., Lee, S. M., & Lin, X. (2018). Interpreter-mediated Cognitive Assessments: Who Wins and Who Loses? *Clinical Gerontologist, 41*(3), 227-236. doi:https://doi.org/10.1080/07317115.2017.1398798 | To explore the experience of interpreter-mediated assessments from the perspectives of clinicians, interpreters and carers. | Using interpreters significantly improved the quality of cognitive assessments of immigrants. | Y | Medium |
| Hashimoto-Govindasamy L.S., Rose V. (2018). An ethnographic process evaluation of a community support program with Sudanese refugee women in western Sydney. *Health promotion journal of Australia, 22*(2), 107-112. doi:https://doi.org/10.1071/HE11107 | To evaluate a Sudanese women’s group exercise program based on a community development strengths-based model. | Participants found the educational components of the program to be effective together with its associated services of transportation, childcare support, and relaxation. | Y | Medium |
| Kalantidou, E. (2018). Design interventions to repair migrants’ mental well-being: The ‘Culture in Mind’ healing space. *Crossings: Journal of Migration & Culture, 9*(2), 133-153. doi:https://doi.org/10.1386/cjmc.9.2.133_1 | To assess the impacts of transforming a service in Brisbane that provided migrant-oriented mental health services into one that included healing processes, intercultural understanding and community empowerment. | The design improved treatment and working conditions for migrant patients and staff. | Y | Medium |
| Karageorge, A., Rhodes, P., & Gray, R. (2018). Relationship and family therapy for newly resettled refugees: An interpretive description of staff experiences. *Australian and New Zealand Journal of Family Therapy, 39*(3), 303-319. doi:https://doi.org/10.1002/anzf.1325 | To assess the Strength to Strength program (STS) which is a counselling service for newly arrived refugees in Sydney, Australia. | The effectiveness of clinical innovations to standard care depended on the use of community groups, the unique role of the bicultural worker, and increased reliance on more directive and practical modes of family therapy. | Y | Lower |
| Kayrouz, R., Karin, E., Staples, L. G., Nielssen, O., Dear, B. F., & Titov, N. (2020). A comparison of the characteristics and treatment outcomes of migrant and Australian-born users of a national digital mental health service. *BMC Psychiatry, 20*(111), 1-13. doi:https://doi.org/10.1186/s12888-020-02486-3 | To compare the clinical outcomes of an Australian national digital mental health service in relation to non-Australian born (migrant) and Australian-born users. | Online treatment was effective in reducing anxiety and depression in both non-Australian born (migrant) and Australian-born users, reflecting the potential of DMHS to reduce barriers to mental health care for migrants. | Y | Medium |
| Kellow, N. J., Palermo, C., & Choi, T. S. (2020). Not Scared of Sugar™: Outcomes of a structured type 2 diabetes group education program for Chinese Australians. *Health and Social Care in the Community, 28*(6), 2273-2281. doi:https://doi.org/10.1111/hsc.13046 | To develop and evaluate a pilot type 2 diabetes group education program aimed for Chinese migrants living in Australia. | The diabetes education program was successful in increasing diabetes self-management skills in the Chinese migrants. | Y | Medium |
| Khan, R., Bedford, K., & Williams, M. (2011). Evaluation of the MindMatters buddy support scheme in southwest Sydney: Strategies, achievements and challenges. *Health Education Journal, 71*(3), 320-326. doi:DOI: 10.1177/0017896911398818 | To assess the strategies, achievements and challenges associated with MindMatters and the views of partner schools of the buddy support scheme. | The programs was effective in empowering students, improving community engagement and building relationships with agencies. | N | Medium |
| Mahimbo A., Seale H., Smith M., & Heywood, A. (2017). Challenges in immunisation service delivery for refugees in Australia: A health system perspective. *Vaccine, 35*(38), 5148-5155. doi:10.1016/j.vaccine.2017.08.002 | To assess access to immunisation services for newly arrived refugees. | Refugees experienced significant barriers in accessing the service. The main challenges impacting on the effectiveness of vaccine coverage for refugees were the inconsistencies associated with the access to program funding and vaccines, insufficient national policy for catch-up vaccination and unclear roles and responsibilities for catch-up vaccination, a deficient central immunisation register and lack of training for general practitioners. | N | Higher |
| Manchikanti, P., Cheng, I.-H., Advocat, J., & Russell, G. (2017). Acceptability of general practice services for Afghan refugees in south-eastern Melbourne. *Australian Journal of Primary Health, 23*(1), 87-91. doi:https://doi.org/10.1071/PY16020 | To assess general practitioner (GP) services as perceived by Afghan refugees in south-eastern Melbourne. | In the view of the participants, the effectiveness of the GP service depended on 1) their ability to receive a detailed clinical assessments, diagnostic investigations and prescriptions at the first consultation, 2) the availability of refugee-friendly staff, and 3) the availability of integrated 'one-stop-shop' GP clinic features. | N | Higher |
| Mander, S., & Miller, Y. D. (2016). Perceived Safety, Quality and Cultural Competency of Maternity Care for Culturally and Linguistically Diverse Women in Queensland. *Journal of Racial and Ethnic Health Disparities, 3*, 83-98. doi:10.1007/s40615-015-0118-7 | To assess maternity care services as experienced by CALD women in Queensland. | The perceived ineffectiveness of maternity care by CALD women in Queensland was due to them perceiving maternity care as delivered by staff lacking technical competence, safety procedures, interpersonal sensitivity, and cultural awareness, leading to CALD women being disadvantaged in receiving maternity care. | N | Higher |
| Maneze, D., Ramjan, L., DiGiacomo, M., Everett, B., Davidson, P. M., & Salamonson, Y. (2018). Negotiating health and chronic illness in Filipino-Australians: a qualitative study with implications for health promotion. *Ethnicity & Health, 23*(6), 611-628. doi:10.1080/13557858.2017.1294656 | To assess services to manage chronic illnesses among Filipino-Australian migrants. | Filipino-Australian migrants were limited in their ability to access services and participate in health promotion initiatives, due to negative experiences arising from language difficulties, lack of social support and persistent cultural issues. | N | Higher |
| Mariño, R., Calache, H., & Morgan, M. (2013). A Community-Based Culturally Competent Oral Health Promotion for Migrant Older Adults Living in Melbourne, Australia. *Journal of the American Geriatrics Society, 61*(2), 270-275. doi: https://doi.org/10.1111/jgs.12078 | To evaluate the effectiveness of an oral hygiene and gingival health intervention delivered to independent-living Italian older adults by a nondental peer educator. | The intervention was effective in increasing self-efficacy among the participant group and reduced gingival bleeding. | Y | Higher |
| Mariño, R., Calache, H., Wright, C., Schofield, M., & Minichiello, V. (2004). Oral health promotion programme for older migrant adults. *Gerodontology, 21*(4), 216-225. doi:10.1111/j.1741-2358.2004.00035.x | To examine the impact of a community-based oral health promotion programme on of older Greek and Italian adults going to community clubs and living in Melbourne. | The community-based oral health promotion programme had positive impacts among the participants in comparison to the control groups. | Y | Higher |
| Mariño, R., Wright, C., Minichiello, V., Schofield, M., & Calache, H. (2005). A qualitative process evaluation of an oral health promotion program for older migrant adults. *Health promotion journal of Australia, 16*(3), 225-228. doi:https://doi.org/10.1071/HE05225 | To evaluate a community-based health promotion program aimed at improving the use of oral health services, oral health knowledge, attitudes, and practices of older Greek and Italian adults living in Melbourne. | Participants perceived the program to be effective and believed the program improved their oral health knowledge and awareness of oral health issues. | Y | Medium |
| Marshall, S., SarahTaki, Love, P., Kearney, M., NancyTam, Sabry, M., . . . Rissel, C. (2021). Navigating infant feeding supports after migration: Perspectives of Arabic and Chinese mothers and health professionals in Australia. *Women and Birth, 34*(4), e346-e356. doi:http://dx.doi.org/10.1016/j.wombi.2020.06.002 | To assess services available for infant feeding among Arabic and Chinese speaking migrant mothers in Australia. | The infant feeding support available for Arabic and Chinese speaking migrant mothers in Australia can improve on their effectiveness by including better support with infant feeding practices and improving cultural sensitivity and cultural relevance of infant feeding programs and child health services. | N | Higher |
| Martin, L., Knight, V., Read, P. J., & McNulty, A. (2013). Fast-track services for all? The preferences of Chinese-, Korean-, and Thai-speaking women attending a sexual health service. *Sexually Transmitted Diseases, 40*(12), 929-932. doi:10.1097/olq.0000000000000055 | To determine the acceptability of a CALD express clinic in Sydney Sexual Health Centre (SSHC), and its individual components, among female Chinese, Korean, and Thai clients. | The express model used at Sydney Sexual Health Centre (SSHC) was not viewed as effective by its CALD client base. | N | Medium |
| Martin, T. J., Butters, C., & Phuong, L. (2017). A two-way street: reciprocal teaching and learning in refugee health. *Australian Health Review, 42*(1), 1-4. doi:https://doi.org/10.1071/AH17055 | To evaluate the Water Well Project, a health promotion charity for individuals of refugee, asylum seeker and migrant backgrounds. | The outcomes of the Water Well Project were positive for session participants, volunteers and referral community organisations. | Y | Lower |
| Mazbouh-Moussa, R., & Ohtsuka, K. (2017). Cultural competence in working with the Arab Australian community: a conceptual review and the experience of the Arab Council Australia (ACA) gambling help counselling service. *Asian J of Gambling Issues and Public Health 7:10, 7*(10), 1-17. doi:https://doi.org/10.1186/s40405-017-0029-0 | To assess gambling counselling services for the Arab community in New South Wales. | The effectiveness of the gambling counselling service for Arab community was reduced by the community’s help seeking behaviour influenced by their cultural beliefs and expectations. | Y | Lower |
| McBride, J., Russo, A., & Block, A. (2016). The Refugee Health Nurse Liaison: a nurse led initiative to improve healthcare for asylum seekers and refugees. *Contemporary Nurse, 52*(6), 710-721. doi:10.1080/10376178.2016.1238774 | To assess asylum seekers and refugees’ access to hospital settings. | The Refugee Health Nurse Liaisons were effective in providing clinical support, advocacy, education, referrals, and both formal and informal capacity building. | Y | Medium |
| Milne, B., Raman, S., Thomas, P., & Shah, S. (2006). Immunisation of refugee and migrant young people: can schools do the job? *Australian and New Zealand Journal of Public Health, 30*(6), 526-528. doi:10.1111/j.1467-842x.2006.tb00780.x | To assess a school-based immunisation service for refugee and migrant young people in Western Sydney attending an Intensive English Centre (IEC) high school. | Low immunisation rates were reported among the refugee and migrant youth attending IEC, reflecting an urgent need for effective and specialised immunisation services for refugee and migrant young people. | Y | Higher |
| Mitchell, J., Kaplan, I., & Crowe, L. (2007). Two cultures: one life. *Community Development Journal, 42*(3), 282–298. doi:https://doi.org/10.1093/cdj/bsl016 | To assess a community capacity-building programme designed for South Sudanese refugees living in an outer western suburb of Melbourne. | The community recovery model was effective. | Y | Lower |
| Momartin, S., Coello, M., Pittaway, E., Downham, R., & Aroche, J. (2019). Capoeira Angola: An alternative intervention program for traumatized adolescent refugees from war-torn countries. *Torture, 29*(1), 85–96. doi:https://doi.org/10.7146/torture.v29i1.112897 | To assess the effectiveness of the STARTTS Capoeira Angola program for participating refugees from Middle Eastern and African countries (aged12-17). | The STARTTS Capoeira Angola program was effective as it delivered a significant decrease in behavioural problems among the participants, improving their interpersonal skills, confidence, respect for self and others, self-discipline, and overall sense of responsibility. | Y | Lower |
| Momartin, S., Miranda, D., Silva, E., Aroche, J., & Coello, M. (2018). Resilience building through alternative intervention: ‘STARTTS “Project Bantu Capoeira Angola”’; on the road to recovery. *Intervention, 16*(2), 154-160. doi:DOI: 10.4103/INTV.INTV_6_18 | To evaluate the impact of Capoeira Angola programme on refugee adolescents. | The Capoeira Angola programme created positive impacts on refugee adolescents improving their resilience, self-esteem, interpersonal relationships and school attendance. | Y | Lower |
| Mozooni, M., Pennell, C. E., & Preen, D. B. (2020). Healthcare factors associated with the risk of antepartum and intrapartum stillbirth in migrants in Western Australia (2005-2013): A retrospective cohort study. *PLoS Medicine, 17*(3), 1-25. doi:10.1371/journal.pmed.1003061 | To assess the impact of access to healthcare services on the risk of stillbirth (SB)—antepartum stillbirth (AnteSB) and intrapartum stillbirth (IntraSB) – among migrant women in Western Australia. | Health care utilisation among migrant women in Western Australia was ineffective as they experienced a high risk of stillbirth due to late commencement of ANC, underutilisation of interpreter services and midwife-only intrapartum care. | N | Higher |
| Newton, D., Keogh, L., Temple-Smith, M., Fairley, C. K., Chen, M., Bayly, C., . . . Hocking, J. (2013). Key informant perceptions of youth-focussed sexual health promotion programs in Australia. *Sexual Health, 10*(1), 47-56. doi:https://doi.org/10.1071/SH12046 | To explore the impediments to effective sexual health promotion programs in Australia in the view of key informants (KI) and suggest strategies to address the barriers. | Fourteen barriers to effective sexual health promotion programs were identified including lack of cultural sensitivity; a failure to acknowledge differences in literacy, knowledge, and language skills; stigma and shame associated with sexual health; and the continued use of programs that lacked inclusivity. | N | Medium |
| Nickerson, A., Byrow, Y., Pajak, R., McMahon, T., Bryant, R., Christensen, H., & Liddell, B. (2020). 'Tell Your Story': a randomized controlled trial of an online intervention to reduce mental health stigma and increase help-seeking in refugee men with posttraumatic stress. *Psychological Medicine, 50*(5), 781-792. doi:10.1017/S0033291719000606 | To evaluate the effectiveness of an online intervention aimed at reducing self-stigma and improving help-seeking behaviour in refugee men. | The online intervention was effective in its focus on self-stigma reduction and improving help-seeking among refugee men. | Y | Higher |
| Nicol, P., Anthonappa, R., King, N., Slack-Smith, L., Cirillo, G., & Cherian, S. (2015). Caries burden and efficacy of a referral pathway in a cohort of preschool refugee children. *Australian Dental Journal, 60*(1), 73-79. doi:https://doi.org/10.1111/adj.12269 | To assess the early caries experience and effectiveness of a community based dental referral pathway for preschool refugees in Western Australia. | The community based dental referral pathway was ineffective in comparison to co-located intersectoral dental screening. | N | Lower |
| Perusco, A., Poder, N., Mohsin, M., Rikard-Bell, G., Rissel, C., Williams, M., . . . Guirguis, S. (2010). Evaluation of a comprehensive tobacco control project targeting Arabic-speakers residing in south west Sydney, Australia. *Health Promotion International, 25*(2), 153-165. doi:10.1093/heapro/daq009 | To evaluate a comprehensive social marketing campaign (SMC) that targeted Arabic-speakers living in south west Sydney. | Comprehensive social marketing campaigns can be an effective method to reduce smoking among targeted CALD populations. | Y | Medium |
| Price, P., Tacey, M., Koufariotis, V., Stramandinoli, D., Vincent, R., Grigg, L., & Zentner, D. (2017). A Contemporary Phone-Based Cardiac Coaching Program: Evolution and Cross Cultural Utility. *Heart, Lung and Circulation, 27*(7), 804-811. doi:http://dx.doi.org/10.1016/j.hlc.2017.07.008 | To evaluate the effectiveness of a phone-based cardiac coaching program to the Greek and Italian populations in comparison to the English cohort. | The phone-based coaching program was effective with CALD patients demonstrating comparable outcomes with the English-speaking cohort. | Y | Higher |
| Quach, A., Laemmle-Ruff, I. L., Polizzi, T., & Paxton, G. A. (2015). Gaps in smiles and services: a cross-sectional study of dental caries in refugee-background children. *BMC Oral Health, 15*, 1-10. doi:http://www.biomedcentral.com/1472-6831/15/10 | To assess and document the oral health status of refugee-background children in Australia and their follow-up at dental services. | The number of African-born children having caries was lesser in comparison to other overseas-born children while phone call reminders was an effective method to keep the attendance rate of refugee-background children high at dental services. | N | Higher |
| Riggs, E., Yelland, J., Shankumar, R., & Kilpatrick, N. (2016). 'We are all scared for the baby': promoting access to dental services for refugee background women during pregnancy. *BMC Pregnancy Childbirth, 16*(12), 1-11. doi:10.1186/s12884-015-0787-6 | To investigate how refugee and asylum seeker community, and dental and maternity care providers in Melbourne perceived maternal oral health, dental priority groups and information provision. | The findings revealed that men and women in the refugee and asylum seeker community perceived dental treatment to be unsafe during pregnancy, and midwives and community participants lacked understanding of the potential impact of poor maternal oral health and lacked awareness of the ‘priority of access’ policy that entitled pregnant women to receive dental care cost-free. | N | Higher |
| Roberts, M., Lobo, R., & Sorenson, A. (2017). Evaluating the Sharing Stories youth theatre program: an interactive theatre and drama-based strategy for sexual health promotion among multicultural youth. *Health promotion journal of Australia, 28*, 30-36. doi:https://doi.org/10.1071/HE15096 | To evaluate the effectiveness of Sharing Stories youth theatre program for multicultural youth. | The program was effective in raising confidence in seeking support about sexual issues, improving sexual health knowledge and improving the attitudes towards sexual health among multicultural youth. | Y | Medium |
| Rodriguez, L. (2013). The subjective experience of Polynesians in the Australian health system. *Health Sociology Review, 22*(4), 411-421. doi:https://doi.org/10.5172/hesr.2013.22.4.411 | To assess Polynesian migrants’ views about health services in Sydney and the Hunter region of New South Wales. | Findings pointed to social disadvantage as a factor impacting on health outcomes, and cultural behaviour and poverty as contributing factors towards obesity-related illness and non-compliance with recommended preventative practices in Polynesian migrants, which reduced the effectiveness of health services for them. | N | Lower |
| Ruwanpathirana, T., Owen, A., Renzaho, A. M., Zomer, E., Gambhir, M., & Reid, C. M. (2015). Can oral vitamin D prevent the cardiovascular diseases among migrants in Australia? Provider perspective using Markov modelling. *Clinical and Experimental Pharmacology and Physiology, 42*(6), 596-601. doi:10.1111/1440-1681.12399 | To understand health care providers' perspective about the effectiveness and cost effectiveness of oral Vitamin D supplementation as a primary prevention strategy for cardiovascular disease among a migrant population in Australia. | Oral Vitamin D supplementation as a primary prevention strategy for cardiovascular disease among a migrant population was cost-effective and reduced non-fatal and fatal cardiovascular outcomes in high-risk migrant populations. | Y | Higher |
| Schulz, T. R., Leder, K., Akinci, I., & Biggs, B.-A. (2015). Improvements in patient care: videoconferencing to improve access to interpreters during clinical consultations for refugee and immigrant patients. *Australian Health Review, 39*(4), 395-399. doi:https://doi.org/10.1071/AH14124 | To assess the acceptability of using an interpreter via videoconferencing compared to on-site or telephone interpreting. | Doctors and patients found using an interpreter via videoconferencing more effective over telephone interpreting. | Y | Lower |
| Schulz, T. R., Richards, M., Gasko, H., Lohrey, J., Hibbert, M. E., & Biggs, B.-A. (2014). Telehealth: experience of the first 120 consultations delivered from a new Refugee Telehealth clinic. *Internal Medicine Journal, 44*(10), 981-985. doi:https://doi.org/10.1111/imj.12537 | To assess the effectiveness of a new telehealth clinic for refugee patients in terms of travel avoided and their demographic and disease profile. | Telehealth using videoconferencing was effective and proved to be a new dimension of care for refugee and immigrant patients settling in regional areas. | Y | Medium |
| Shafiei, T., Small, R., & McLachlan, H. (2015). Immigrant Afghan women’s emotional well-being after birth and use of health services in Melbourne, Australia. *Midwifery, 31*(7), 671-677. doi:https://doi.org/10.1016/j.midw.2015.03.011 | To assess the impact of health services on the emotional well-being and postnatal depression of immigrant Afghan women. | Immigrant Afghan women experienced emotional distress due to isolation, lack of support and their life circumstances, but did not believe health professional have the capacity to provide any relief or support, which led to ineffective utilisation of health services by them. | N | Medium |
| Shanmugasundaram, S., & O'Connor, M. (2009). Palliative care services for Indian migrants in Australia: Experiences of the family of terminally Ill patients. *Indian Journal of Palliative Care, 15*(1), 76-83. doi:10.4103/0973-1075.53589 | To examine the access to palliative care services for Indian migrants in Australia | The effectiveness of palliative care services was reduced for Indian patients due to the difficulties their families experienced in accessing palliative care services because of the nature of Indian support systems, cultural issues, and caring experiences. | N | Higher |
| Shaw, J. M., Shepherd, H. L., Durcinoska, I., Butow, P. N., Liauw, W., Goldstein, D., & Young, J. M. (2016). It’s all good on the surface: care coordination experiences of migrant cancer patients in Australia. *Supportive Care in Cancer, 24*, 2403–2410. doi:10.1007/s00520-015-3043-8 | To assess migrant patients’ experience of care coordination to inform the development of items for inclusion in a cancer care coordination questionnaire that’s specific to their cultural needs. | The four significant themes related to the effectiveness of cancer care coordination were the impact of language on understanding and information access, the role of interpreters, access to services and understanding the roles and responsibilities of the team. | Y | Higher |
| Shaw, J., Butow, P., Sze, M., Young, J., & Goldstein, D. (2013). Reducing disparity in outcomes for immigrants with cancer: a qualitative assessment of the feasibility and acceptability of a culturally targeted telephone-based supportive care intervention. *Supportive Care in Cancer, 21*, 2297–2301. doi:10.1007/s00520-013-1786-7 | To assess telephone-based supportive care interventions for Chinese or Arabic cancer patients. | The intervention delivery and support provided in patients’ language was viewed favourably while cultural sensitivities included confidentiality about the illness. | N | Medium |
| Sheikh, M., & MacIntyre, C. R. (2009). The impact of intensive health promotion to a targeted refugee population on utilisation of a new refugee paediatric clinic at the children's hospital at Westmead. *Ethnicity & Health, 14*(4), 393-405. doi:10.1080/13557850802653780 | To assess the impact of a targeted new health service delivered to the refugee population recently settled in Sydney. | The targeted health promotion campaign was effective in increasing the participation of the targeted communities in comparison with the non-targeted communities. | Y | Medium |
| Sievert, K., O’Neill, P., Koh, Y., Lee, J. H., Dev, A., & Le, S. (2018). Engaging new refugee in Australian communities at risk for chronic hepatitis B infection into care: A peer-educator intervention. *Health and Social Care in the Community, 26*(5), 744-750. doi: https://doi.org/10.1111/hsc.12602 | To assess the effectiveness of a peer-education Chronic Hepatitis B (CHB) intervention delivered to the three communities of Afghan, Rohingyan, and Sudanese communities as radio programmes and community forums in their own language. | A peer-educator approach was cost-effective and successful mostly for Afghan and Rohingya communities while engaging the South Sudanese community proved challenging. | Y | Lower |
| Slewa-Younan, S., McKenzie, M., Thomson, R., Smith, M., Mohammad, Y., & Mond, J. (2020). Improving the mental wellbeing of Arabic speaking refugees: an evaluation of a mental health promotion program. *BMC Psychiatry, 20*(314), 1-13. doi:https://doi.org/10.1186/s12888-020-02732-8 | To evaluate the effectiveness of a culturally tailored mental health promotion program for Arabic-speaking refugees. | Inclusion of mental health literacy in a culturally tailored mental health program can increase positive mental health outcomes for Arabic-speaking refugees resettled in a Western nation. | Y | Lower |
| Small, R., Rice, P. L., Yelland, J., & Lumley, J. (1999). Mothers in a New Country: The Role of Culture and Communication in Vietnamese, Turkish and Filipino Women's Experiences of Giving Birth in Australia. *Women & Health, 28*(3), 77-101. doi:10.1300/J013v28n03_06 | To explore how Vietnamese, Turkish and Filipino women viewed their maternity care and their experiences of early motherhood. | Communication challenges due to language lessened effectiveness of care while the lack of cultural awareness of the caregivers was perceived as secondary to the issues of their unsupportive behaviour and rushed care. | N | Lower |
| Smith, R., & Gallego, G. (2021). Parents’ ability to access community health occupational therapy services in a disadvantaged area: A proof of concept study. *Australian Occupational Therapy Journal, 68*, 54-64. doi:10.1111/1440-1630.12699 | To identify barriers and enablers to accessing child and family occupational therapy services in a disadvantaged area focusing on two predominant language groups of Arabic speaking and Bangladeshi families in three suburbs in New South Wales. | Outreach occupational therapy services was effective in improving access to allied health services for disadvantaged families with young children while barriers were identified as lack of awareness, and transport and logistical issues. | N | Medium |
| Stapleton, H., Murphy, R., Correa-Velez, I., Steel, M., & Kildea, S. (2013). Women from refugee backgrounds and their experiences of attending a specialist antenatal clinic. Narratives from an Australian setting. *Women and Birth, 26*(4), 260-266. doi:https://doi.org/10.1016/j.wombi.2013.07.004 | To investigate if maternity experience of refugee women attending a specialist antenatal clinic in a tertiary Australian public hospital can be improved. | The effectiveness of maternity care for refugee women can be improved through the delivery of comprehensive and culturally sensitive maternity care which, however, require intensive resources. | Y | Medium |
| Steel, Z., Mcdonald, R., Silove, D., Bauman, A., Sandford, P., Herron, J., & Harry Minas, I. (2006). Pathways to the First Contact with Specialist Mental Health Care. *Australian & New Zealand Journal of Psychiatry, 40*(4), 347-354. doi:10.1080/j.1440-1614.2006.01801.x | To assess the access to mental health care and the degree to which cultural background, illness type, severity and service-related variables influenced the decisions to access care. | While social and cultural factors influenced the mental health patients’ decision to consult a range of professionals, social and cultural factors did not impact on the effectiveness the role played by public mental health services as no patient delayed their decision to attend public mental health services based on gender, social support, ethnicity or English fluency. | N | Medium |
| Sulaiman, N., Hadj, E., Hussein, A., & Young, D. (2013). Peer-Supported Diabetes Prevention Program for Turkish- and Arabic-Speaking Communities in Australia. *ISRN Family Medicine, 2013*, 1-6. doi:http://dx.doi.org/10.5402/2013/735359 | To develop and evaluate a culturally sensitive peer-supported diabetes education program for the prevention of type 2 diabetes in high-risk middle-aged Turkish- and Arabic-speaking people and evaluate its effectiveness. | A short diabetes prevention program delivered by bilingual peers was effective in improving diabetes awareness, changing lifestyle behaviour and reduction in body weight within 3 months of the intervention. | Y | Lower |
| Sypek, S., Clugston, G., & Phillips, C. (2008). Critical health infrastructure for refugee resettlement in rural Australia: case study of four rural towns. *Australian Journal of Rural Health, 16*(6), 349-354. doi:10.1111/j.1440-1584.2008.01015.x | To examine the impact of regional refugee resettlement on rural health services and identify critical health infrastructure required for refugee resettlement. | Health services in regional areas were less effective in treating refugees resettled in regional areas due to the lack of practitioners, high levels of turnover of health care staff and the lack of specialist knowledge among health care workers to treat refugees. | N | Medium |
| ThuyTrinh, L. T., Stephenson, J., & Vajda, J. (2011). Radio campaign to promote quality use of medicines among Italian, Mandarin and Cantonese speaking seniors in Australia. *Health Promot J Austr, 22*(1), 51-56. doi:10.1071/he11051 | To evaluate the effectiveness of a radio campaign in promoting the quality use of medicine (QUM) among Italian, Mandarin and Cantonese-speaking seniors. | The radio campaign was effective in increasing awareness and knowledge of quality use of medicine among the seniors studied, but the effectiveness varied between language groups. | Y | Higher |
| van Gemert, C., Wang, J., Simmons, J., Cowie, B., Boyle, D., Stoove, M., . . . Hellard, M. (2016). Improving the identification of priority populations to increase hepatitis B testing rates, 2012. *BMC Public Health, 16*, 1-8. doi:10.1186/s12889-016-2716-7 | To assess access to a screening intervention to improve CHB (chronic hepatitis B) diagnosis among priority populations in Melbourne, Australia. | The intervention proved effective at identifying Asian-born patients and patients of Asian ethnicity who may be at increased risk of CHB but failed to improve HBV testing behaviour in this priority population. | N | Higher |
| van Wyk, S., Schweitzer, R., Brough, M., Vromans, L., & Murray, K. (2012). A longitudinal study of mental health in refugees from Burma: The impact of therapeutic interventions. *Australian and New Zealand Journal of Psychiatry, 46*(10), 995–1003. doi:10.1177/0004867412443059 | To examine the impact of a therapeutic intervention for people from refugee backgrounds within a naturalistic setting. | The intervention was able to create a significant reduction of symptoms of post-traumatic stress disorder, anxiety, depression and somatisation among the participants. | Y | Lower |
| Vaughan, L., Schubert, L., Mavoa, H., & Fa'avale, N. (2017). 'Hey, We Are the Best Ones at Dealing with Our Own': Embedding a Culturally Competent Program for Māori and Pacific Island Children into a Mainstream Health Service in Queensland, Australia. *Journal of Racial and Ethnic Health Disparities, 5*(3), 605-616. doi:10.1007/s40615-017-0406-5 | To evaluate Good Start Program (GSP), which aims at preventing chronic disease among Maori and Pacific Island (MPI) communities living in the state of Queensland, Australia. | Culturally tailored programs delivered by multicultural health workers (MHWs) were effective in improving health and healthy behaviours among targeted groups. | Y | Lower |
| Wamwayi M.O.; Cope V.; Murray M. (2019). Service gaps related to culturally appropriate mental health care for African immigrants. *International Journal of Mental Health Nursing, 28*(5), 1113-1121. doi: https://doi.org/10.1111/inm.12622 | To assess the access to hospital inpatient mental health for African immigrants from the perspective of staff. | Inadequate interpreter services, lack of cultural awareness staff training, lack of organisation link with other services, unmet spiritual needs, use of staff/families as interpreters, culturally inappropriate information, and lack of or inadequate culturally appropriate policies and framework were identified as the service gaps that limited the effectiveness of the service. | N | Higher |
| Willey, S. M., Gibson-Helm, M. E., Finch, T. L., East, C. E., Khan, N. N., Boyd, L. M., & Boyle, J. A. (2020). Implementing innovative evidence-based perinatal mental health screening for women of refugee background. *Women and Birth, 33*(3), e245-e255. doi:https://doi.org/10.1016/j.wombi.2019.05.007 | To evaluate a perinatal mental health screening program for refugee women. | The perinatal mental health screening program was effective and feasible in the point of view of health professionals. | Y | Lower |
| Williams, A., Manias, E., Liew, D., Gock, H., & Gorelik, A. (2012). Working with CALD groups: testing the feasibility of an intervention to improve medication self-management in people with kidney disease, diabetes, and cardiovascular disease. *Renal Society of Australasia journal, 8*(2), 62-69. doi:https://dro.deakin.edu.au/view/DU:30076303 | To examine the effectiveness of an intervention aimed at addressing medication mismanagement among CALD groups and report on the challenges associated with studying CALD groups. | No significant differences were discernible of medication self-efficacy between the intervention and control groups on non-CALD participants at three, six and 12 months post-baseline. | N | Higher |
| Wollersheim, D., Koh, L., Walker, R., & Liamputtong, P. (2013). Constant connections: Piloting a mobile phone-based peer support program for Nuer (southern Sudanese) women. *Australian Journal of Primary Health, 19*(1), 7-13. doi:https://doi.org/10.1071/PY11052 | To assess a mobile phone-based peer support aiming to improve the settlement experiences of a group of nine Nuer refugee women in Melbourne, Australia. | The mobile phone-based peer support was effective in recreating the feeling of community through connecting geographically separated refugee women. | Y | Lower |
| Woodland, L., Kang, M., Elliot, C., Perry, A., Eagar, S., & Zwi, K. (2016). Evaluation of a school screening programme for young people from refugee backgrounds. *Journal of Paediatrics and Child Health, 52*, 72-79. doi:https://doi.org/10.1111/jpc.12989 | To describe and evaluate the Optimising Health and Learning Program as a health delivery service to newly arrived refugee children. | The program was effective in detecting health conditions of refuge children, linking newly arrived students and their families with primary health care, and coordinating care across primary health and specialist services. | Y | Medium |
| Woolfenden, S., Posada, N., Krchnakova, R., Crawford, J., Gilbert, J., Jursik, B., . . . Kemp, L. (2014). Equitable access to developmental surveillance and early intervention – understanding the barriers for children from culturally and linguistically diverse (CALD) backgrounds. *Health Expectations, 18*(6), 3286-3301. doi:https://doi.org/10.1111/hex.12318 | To assess CALD people’s access to developmental surveillance (DS) and early intervention services in south-eastern Sydney, Australia. | The factors influencing the effectiveness of DS access were resources, extended family and social support, information availability, competing needs, complex service pathways and community engagement. | N | Medium |
| Xiao, L., Habel, L., & Bellis, A. D. (2015). Perceived Challenges in Dementia Care by Vietnamese Family Caregivers and Care Workers in South Australia. *Journal of Cross-Cultural Gerontology, 30*(3), 333-352. doi:10.1007/s10823-015-9264-y | To assess dementia care services for Vietnamese families. | Family stigma was a significant barrier reducing effectiveness of dementia care for Vietnamese care workers when providing dementia care while both Vietnamese family caregivers and Vietnamese care workers showed differing perspectives of family stigma. | N | Higher |
| Yelland, J., Mensah, F., Riggs, E., McDonald, E., Szwarc, J., Dawson, W., . . . Brown, S. (2020). Evaluation of systems reform in public hospitals, Victoria, Australia, to improve access to antenatal care for women of refugee background: An interrupted time series design. *PLoS Medicine, 17*(7), 1-20. doi:https:// doi.org/10.1371/journal.pmed.1003089 | To assess refugee women’s access to hospital-based antenatal care. | Hospital-based antenatal care during the period of health system reforms was ineffective for refugee women as a steady decrease was observed in the proportion of women having their first hospital visit at less than 16 weeks’ gestation, which was prevalent mostly among refugee women. | Y | Higher |
| Young, M. K., McCall, B. J., & Heel, K. (2010). The impact of pre-departure screening and treatment on notifications of malaria in refugees in south-east Queensland. *Communicable diseases intelligence, 34*(1), 37-40. | To assess a program of pre-departure screening of malaria among refugees. | The national policy change on pre-departure screening was effective as the greatest proportion and number of notifications that was recorded in 2005 decreased in the subsequent 2 years, demonstrating a statistically significant decline. | Y | Lower |
